# Coping with Examination Stress: An Emotion Analysis

**DOI:** 10.3390/s24134297

**Published:** 2024-07-02

**Authors:** Spyros Avdimiotis, Ioannis Konstantinidis, George Stalidis, Dimitrios Stamovlasis

**Affiliations:** 1 Department of Organizations Management and Tourism, Faculty of Economy and Management, International Hellenic University, 57001 Thessaloniki, Greece; soga@ihu.gr (S.A.); stalidgi@ihu.gr (G.S.); 2 Department of Philosophy and Education, Aristotle University of Thessaloniki, 54124 Thessaloniki, Greece; stadi@edlit.auth.gr

**Keywords:** stress, network analysis, exams, EEG, examination stress

## Abstract

Stress is an important factor affecting human behavior, with recent works in the literature distinguishing it as either productive or destructive. The present study investigated how the primary emotion of stress is correlated with engagement, focus, interest, excitement, and relaxation during university students’ examination processes. Given that examinations are highly stressful processes, twenty-six postgraduate students participated in a four-phase experiment (rest, written examination, oral examination, and rest) conducted at the International Hellenic University (IHU) using a modified Trier protocol. Network analysis with a focus on centralities was employed for data processing. The results highlight the important role of stress in the examination process; correlate stress with other emotions, such as interest, engagement, enthusiasm, relaxation, and concentration; and, finally, suggest ways to control and creatively utilize stress.

## 1. Introduction

In antiquity, ancient Greek philosophers defined the concept of stress by relating it to the utterance and structure of speech and its negative effects on the human body [1]. Stress has always been, and remains, a subject of study in several scientific fields, making it important for researchers to determine its role in addressing the homeostatic changes that it causes. One definition of stress paints it as an organism’s specific response to any kind of challenge [2]. Unlike anxiety, which is a physiological response, stress is a behavioral phenomenon caused by either external factors (i.e., exogenous factors, strong sound stimuli, time constraints, and traumatic experiences in general) or internal factors (i.e., endogenous processes that may occur in an organism and cause stressful reactions) [3]. Generally, faced with a stressful stimulus, an organism perceives that the demands exceed the available resources, activating an alarm state that causes a homeostatic change. The organism itself assesses the situation and response; thus, each individual responds differently to each stressor.

In learning organizations, such as universities, stress is an integral part of students’ lives [4]. Therefore, stress strongly affects their everyday life and is closely related to their performance, especially if it is intense and repetitive [5]. In some extreme cases, it is even associated with mental health disorders and leads to students dropping out [6,7,8,9] due to the pressure caused by assignments that must be handed in, deadlines that must be met, the need for punctuality in class attendance, and, most importantly, exams. Exams are probably the most stressful process that every student must face and successfully complete in order to progress through the stages of their studies and become a member of the international scientific community. Their performance during the examination process is inextricably linked to stress intensity, often determining their success [10]. The knowledge acquired through the educational process and their preparational efforts must be transferred as accurately as possible to performance in an examination in order to complete it successfully. In other words, the transferred knowledge, which has been stored in their long-term memory, must be recalled in the best possible way during the examination process, disregarding any negative feelings that may hinder it. Stress is one of the emotions that, when intense, can potentially affect or even deactivate specific brain areas and start other background processes, to ensure the organism’s survival and allow it to feel safe by successfully responding to this emotion [11,12,13].

For this reason, depending on its intensity and duration, stress may positively or negatively affect an individual’s performance, leading to the conclusion that there are two basic forms of stress: creative (eustress) and destructive (distress) stress. Selye (1956, 1983) [14,15] introduced the term “stress” to the scientific community, distinguishing the positive and negative effects that it may have. The extent to which stress can affect students’ individual performance is related to its ability to significantly affect an individual’s memory and learning functions [16]. Memory comprises a set of interdependent brain systems of different behaviors that determine the separation of the types of information received by an individual, the storage time of the incoming information, its quantification and encoding, and the processes enabling its retrieval [17]. It is generally accepted that there are two major forms of memory: (i) short-term and (ii) long-term memory. There are two types of long-term memory: declarative and non-declarative. The former comprises episodic and semantic memory, and the latter consists of procedural, perceptual, classical, conditioning, and non-associative learning [18]. The stage or process of memory formation consists of three parts: encoding, consolidation, and retrieval [19]. First, stimuli are captured as small representations, so that the brain can associate them with a memory trace, and then the stored information is retrieved whenever requested. Sousa (2022) [18] supports the notion that stress affects declarative and non-declarative memory. The former is associated with explicit knowledge, including episodic, significant, and short-term memory, while the latter is associated with intangible knowledge, including procedural memory, representational memory, and perception [19].

In recent years, stress and emotions in general have been measured with non-invasive methods used by various sciences such as psychology, robotics, neuroscience, and brain science. These methods are usually utilized to analyze, explain, and identify the effects of each science. A portable electroencephalography (EEG) device is a widely used technique for measuring and analyzing emotions. Delving into the literature, we can address several published research cases where EEG devices have been used as a primary experimental instrument to measure emotions and, especially, stress [20,21,22,23]. The present study used EEG devices to identify the contribution of stress to knowledge transfer and, more specifically, its role in the examination performance of university students, affecting both declarative and non-declarative memory, since several research findings strongly indicate a correlation between brain cognitive activity and a reduction in alpha-band activity [24]. Neurophysiological measurements (NPMs), such as electroencephalography (EEG) and electrodermal activity (EDA), are exciting, effective, and ground-breaking ways of measuring a wide range of emotions, including stress [25], providing interesting and spontaneous data inputs [26].

**Figure 1:**
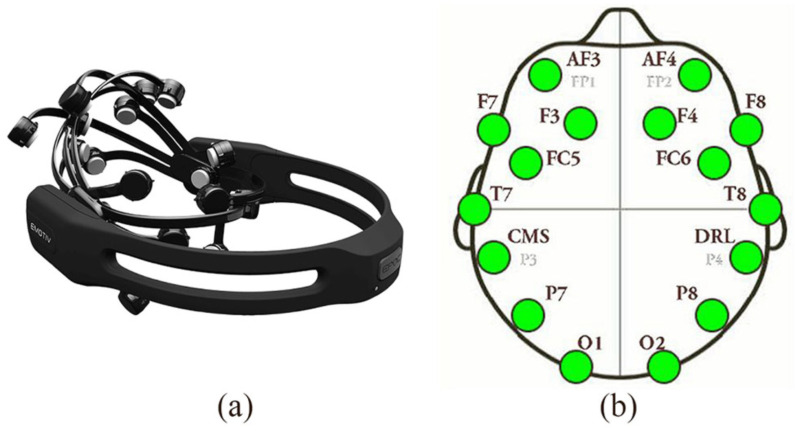
(**a**) Emotiv Epoc+ headset; (**b**) spatial mapping of the electrodes on the scalp (F—frontal, P—parietal, O—occipital, T—temporal, FC—front central, AF—anterio frontal, CMS—common mode sense, DRL—driven right leg) [25].

However, to measure emotions, there must be a stimulus leading to their induction. In this study, instantaneous acute stress was induced in the subjects via a protocol based on the method first used by Trier (the Trier Social Stress Test) [27]. The portable Emotiv Epoc+ EEG device (see Figure 1a) was used to perform the experiment, observe brain activity, and collect data, allowing the researchers to draw useful inferences. The Emotiv EEG device measured brain activity by collecting data from fourteen sensors (see Figure 1b). The data were processed via an application programming interface (API) in Emotiv. The collected values of the emotions, as shown in Figure 3, were considered and used as time-series datasets in EmotivPRO v.3.8.0.532 software to indicate a further analysis after pre-processing and epoching into smaller time frames, according to the phases of the experimental procedure.

The data analysis enabled the creation of a network of emotions for each person, which related to the experimental process. The network formed for each individual was created using JASP v.18.1.0 software with the time-series data for each emotion. Recent research has shown that brain networks can be created by studying and exploring the correlations between EEG channels [28,29]. Each channel represents a node in the network; this process is referred to as brain network modeling. This research attempted to analyze the network of emotions captured via the EEG device based on this methodological approach. The emotions that were analyzed and captured, besides the dominant one of stress, were engagement, excitement, interest, focus, and relaxation. Our work focused on studying the correlations between the emotions forming the network, particularly the correlations between stress and the others, and determining the role of each in the network via their k-centricity values, the concept of which is described below.

Given the valid measurements reflecting the corresponding emotional states [30], the variables under study were constructed at the psychological level and could have been examined via traditional psychometric approaches. Nevertheless, considering psychological phenomena from a network perspective [31,32], a network analysis was proposed and applied to the current empirical data. In such networks, nodes represent psychological variables connected by undirected edges indicating conditional dependence. This analytical method has gained considerable attention in the contemporary literature, because networks realistically represent the relationships between psychological constructs [33] that coexist and coevolve over time.

## 2. Materials and Methods

### 2.1. Data-Processing Methodology

The research team successfully combined common frequency-based EEG data-processing methods with statistical analysis based on networks and centralities of emotions to formulate the innovative approach used in this study. The researchers based their inferences on frequency patterns and reinforced them using tools and methods for statistical analysis. In the experimental data acquisition process, the 14 sensors of the Emotiv Epoc+ device captured each person’s brain activity. A set of frequencies for each emotion was collected via further processing using the company’s software (API). The emotions were pre-established using the EEG device official software (EmotivPRO v.3.8.0.532), produced in the US by Emotiv company (San Francisco, CA, USA); thus, no further processing took place. Based on the data exported by EmotivPRO v.3.8.0.532 (as shown in Figure 3), a value (for each emotion) was given every 10 seconds, depicting the course of emotions during the 40-minute experiment. These were time-series data, since they referred to factor measurements for the entire duration of the experimental process, considered as signals ensuing from the corresponding sources, which were correlated. The values for each emotion were exported to a .csv file, which was processed for each individual, to enable further statistical analysis. The network representation (for each emotion) was constructed using the correlation matrix. Furthermore, a network analysis was conducted, which, via centrality measures, revealed the most important, crucial, or influential nodes (emotions) acting under the experimental conditions.

Regarding the validity of the device and the data acquired, devices such as the Emotiv EPOC portable EEG have been widely used in emotion studies and are, hence, validated and highly reliable for measuring emotions. Zabcikova (2018) [34] used this EEG device to measure performance metrics in relation to visual and auditory stimuli, and Ergan et al. (2019) [35] used the same device to measure stress in architectural virtual environments. Finally, Osornio Garcia et al. (2023) [36] used the same portable EEG and assigned separate values to each emotion to analyze participants’ emotional responses while wandering through a department store. Their datasets consisted of these values and were used to analyze the emotional states and observe the responses of each person during the experimental procedure.

### 2.2. Participants

Twenty-six IHU postgraduate students who volunteered and had no record of psychiatric or neurological illness, or other serious physical illness, participated in this research. Individuals with drug or alcohol addiction issues, people with metal implants in their bodies, and those regularly taking medication were excluded. The participants signed a consent form ensuring anonymity in the personal data management process. Consent was also obtained from the Ethics Committee of the International Hellenic University, which adheres to the Helsinki Declaration.

### 2.3. Instrument Validation

The Emotiv model EPOC+ EEG device used in this study has been validated via its extensive use in several experimental procedures, providing acceptable precision. EPOC+ has been proven to be effective in emotion-mapping studies—specifically, in an experiment where participants were shown images from the International Affective Picture System (IAPS) database, and their emotional responses were recorded and compared with the values provided by the headset’s API. The results demonstrated a high classification accuracy of 85%, indicating the reliability of the emotional estimations provided by Emotiv EPOC+ for use in real-time applications [30]. Williams et al. (2020) used the EPOC EEG device as a reference for researchers seeking cost-effective EEG solutions and to inspire future research using portable EEG devices [37]. Furthermore, the device has been used to explore the advancements in BCI research, including the ability to decode human speech and control brain activity, e.g., to identify emotional parameters using brain signals captured via the Emotiv Epoc+ Neuroheadset [38]. The device has also been used to classify emotional states using machine learning techniques. By analyzing the data, researchers found that they contained sufficient information to differentiate between various emotional states, with different subjects exhibiting varying accuracies [39]. In addition, the device was applied to analyze subjects’ emotions while viewing emotion-evoking images. Concentration and anxiety levels were calculated from brain wave frequencies to determine the participants’ emotions based on these metrics. Specific brain activity patterns were associated with different emotions, and changes in concentration and anxiety levels were observed in response to various images, indicating distinct emotional responses. The study suggested EEG signals’ potential to detect emotional changes [40]. Furthermore, EEG signals were used to explore the classification accuracy of an EEG-based stress recognition system using the Stroop color–word and mental arithmetic tests as stressors. The system achieved a 75% accuracy in classifying the stress levels induced by the two tests upon analyzing the EEG signals and implementing different time windows for stress detection [41]. In addition, an emotion recognition system for emotion classification was presented using EEG signals from the Emotiv EPOC headset while viewing an emotional movie [42]. As observed, the Emotiv Epoc+ device is widely used by the scientific community, and the measurements that it provides are reliable and can be used in further analysis.

Via the Emotiv software (EmotivPRO), the researcher can either separately download the file with the data values concerning each electrode or obtain the values of each emotion separately, as assigned by the software and recorded every 10 s. These are time-series data, since they represent the electrode values translating brain activity, and the device’s API processes these signals in real time to estimate various cognitive states, such as excitement (arousal), interest (valence), stress (frustration), engagement, attention (focus), and meditation (relaxation) [30,43]. The API employs two libraries: EmoEngine, which stores signals from the EEG channels, and EmoKey, which processes these signals to provide numerical values for each channel. These values are normalized between 0 (lower intensity) and 1 (higher intensity). The values are separately assigned for each emotion via this approach, as shown in Figure 3.

Regarding the sensors’ topology, the employed electroencephalograph consists of 14 electrodes placed in the following areas, as can be seen in Figure 1b:

- F3, F4, AF3, AF4, F7, and F8 visualize the activity of the frontal lobe;
- T7, T8, FC5, and FC6 represent the imaging activity in the temporal lobes;
- P7 and P8 represent the imaging activity in the parietal lobes;
- O1 and O2 represent the activity in the occipital lobes.

The device had a sampling rate between 128SPS and 256SPS and a bandwidth of 0.2–45 HZ, with digital notch filters at 50 Hz and 60 Hz. It was wirelessly connected to a computer on which the Emotiv Pro v.3.8.0.532, Emotiv BCI v.4.3.0.290, and Emotiv BrainVIZ v.4.3.0.146 software had been installed in order for the device to work properly and obtain data [44]. The images below show the Emotiv Epoc+ headset and the spatial mapping of the electrodes on the scalp.

The participants were given specific instructions to obtain the best data from the EEG device, such as not to use haircare products, to remove metal objects (i.e., earrings or necklaces), to keep their heads as steady as possible, and to switch off their mobile phones to avoid interfering with the data transmission between the EEG device and the computer.

### 2.4. Procedure

**Figure 2:**
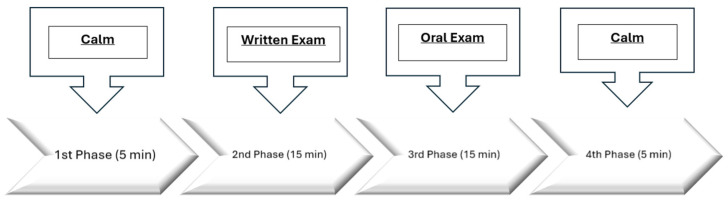
Stages of the experimental procedure.

A modified protocol based on Trier’s Social Stress Test (TSST) was implemented to induce stress and divided into four distinct stages that are incorporated into the final examination of the ‘Organizational Management’ course. The course is included in IHU’s 3rd-semester curriculum for postgraduate studies at the Department of Organization Management, Marketing, and Tourism. Participants were informed of the conditions of the written and oral examination process. Students had to complete a 20-question multiple-choice test in 15 minutes, followed by a 15-minute oral examination. Thus, as stipulated by the protocol, each volunteer appeared at a predetermined date, time, and place before a panel of three people (a professor and two researchers). The duration of the whole examination process was 30 minutes. Five minutes of calm before and after the examination were added, leading to an overall process duration of forty minutes (see Figure 2).

The stages of this research protocol were as follows:

- First, the volunteers came into the room and were given instructions. Afterward, the electroencephalogram (EEG) device and wearable (E4) were applied, and a resting stage began, where each subject sat comfortably in a chair and was asked to close his/her eyes and relax for approximately five minutes. At this stage, baseline measurements were taken, based on which any changes in emotions observed in the second and third stages of the experimental procedure were observed.
- In the second stage, the written examination of the volunteers began, where they were given an exam paper with multiple-choice questions to be answered within fifteen minutes.
- In the third stage, the volunteers were subjected to an oral examination in front of a three-member committee panel for fifteen minutes.
- In the fourth and final stage, the volunteers calmed down for five minutes.

The experimental protocol consisted of four phases. The first was a five-minute phase, the following two were fifteen-minute phases, and the last was another five-minute phase. Distinctive marks were input into the software at the end of each phase. Therefore, each person had four distinctive time-series datasets (epochs), one for each phase.

Figure 2 shows the steps of the experimental methodology, starting with the first stage of the calming phase, followed by the written test and then the oral test. Finally, the protocol was completed with the final calming phase.

### 2.5. Analytical Framework

The data were prepared and integrated in real time. The researchers created the timestamps during the experimental procedure, and the measurements obtained during the first stage were taken as the baseline. The Emotiv software provided the emotion values assigned by the software every 10 s, stored in .csv file format.

The Emotiv software provided the emotion measurements as a separate time series for each emotion, which were further analyzed to create a network of emotions. The six emotions measured, the main one being stress, are described by the creators of Emotiv as follows [43]:

○ Stress: Stress is a measure of human comfort during a challenge that a person must face. High stress can result from an inability to complete a difficult task, feeling overwhelmed, and a fear of negative consequences from potential failure in the task to be performed. In general, a low–moderate level of stress can improve productivity, while a higher level tends to be destructive and can have long-term consequences for health and wellbeing.
○ Engagement: Engagement is experienced as alertness and consciously directing attention toward task-related stimuli. It measures the level of absorption at a point in time and is a mixture of attention and concentration that contrasts with boredom. Engagement is characterized by increased physiological arousal and more beta waves alongside attenuated alpha waves. The greater the attention, focus, and workload, the greater the value of this factor represented by the EEG software (EmotivPRO v.3.8.0.532).
○ Excitement: Excitement is a positive feeling of physiological arousal. It is characterized by activation in the sympathetic nervous system, producing several physiological responses, including pupil dilation, sweat gland stimulation, increased heart rate and muscle tension, blood diversion, and the inhibition of digestive system functions. In general, the greater the increase in physiological stimulation, the greater the value of this factor reflected in the EEG software. Excitement detection is set up to capture values that reflect short-term changes in excitement over periods as short as a few seconds, as reported by Emotiv.
○ Focus: Focus or attention (as it was recently renamed by Emotiv) measures sustained attention to a particular task. Focus measures the depth of attention and the frequency of switching attention between tasks. Frequent switching and difficult tasks can lead to low values for this factor, indicating poor focus and distraction.
○ Interest: Interest represents the degree of attraction or aversion to the current stimuli, environment, or activity and is usually referred to as valence. Low interest scores indicate a strong aversion to the task, and high interest indicates a strong desire for the task, while mid-range scores indicate neither desire nor aversion to the activity.
○ Relaxation: This factor indicates an individual’s ability to minimize their work rate and recover after strong concentration levels.

**Figure 3:**
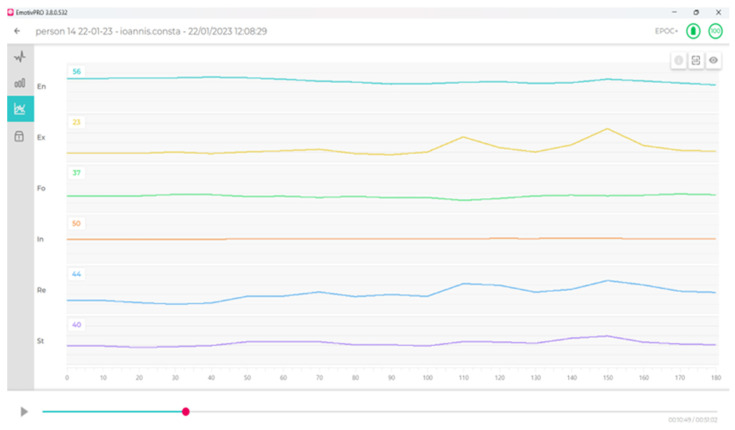
Representation of the six emotions that EmotivPRO software v.3.8.0.532 measures on a scale of 1–100: engagement (En), excitement (Ex), focus (Fo), interest (In), relaxation (Re), and stress (St).

**Figure 4:**
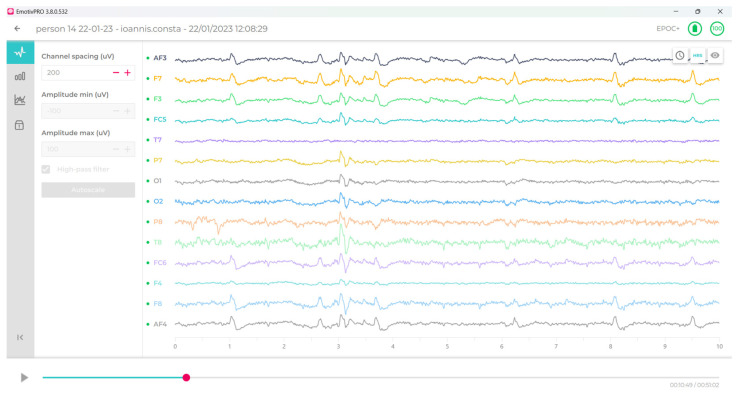
Illustration of the 200 µV RAW electroencephalography (EEG) diagram for 14 electrodes with sufficient contact quality (F—frontal, P—parietal, O—occipital, T—temporal, FC—front central, AF—anterio frontal).

Figure 3 below illustrate the data visualizations of the six emotions—engagement (En), excitement (Ex), focus (Fo), interest (In), relaxation (Re), and stress (St)—in real time during the experimental procedure, as provided by the Emotiv Pro software. Thus, the researchers could watch real-time emotional states throughout the experimental process in either the resting or testing phase. Also, Figure 4 illustrate the 200 µV RAW electroencephalography (EEG) diagram for 14 electrodes with sufficient contact quality.

Each participant’s performance in the experimental procedure was expressed by the mark obtained in the examination procedure (experimental procedure), which was separated into written and oral parts. The written examination procedure consisted of fifteen questions of graded difficulty. In the oral examination procedure, a comprehensive analysis of the course under examination, with an evaluation of the examinee, was carried out via an in-depth discussion with questions of graded difficulty. Performance was correlated with the stress factor and the other measurable factors to draw firm conclusions regarding the knowledge transfer process and the influencing factors.

### 2.6. Data Analysis

**Figure 5:**
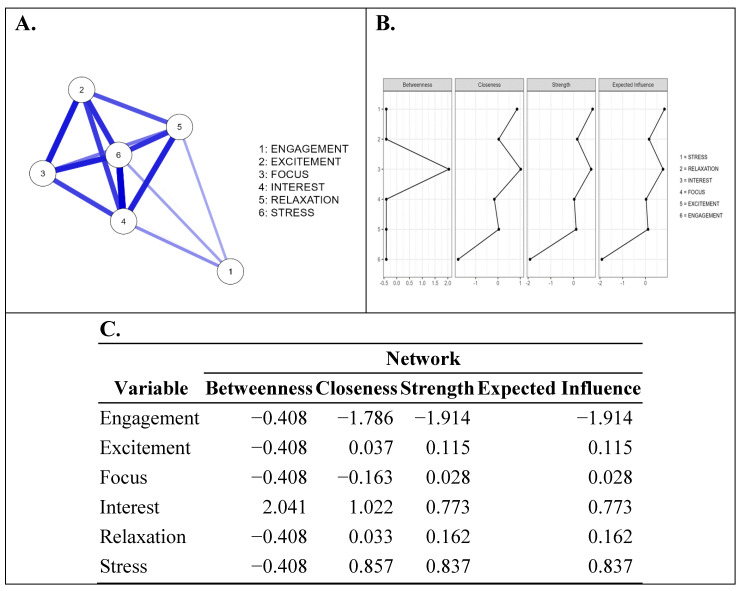
Emotion network analysis per person. (**A**) Network structure of emotions. Blue edges represent positive correlations. The thickness of the edge reflects the magnitude of the correlation. Cut-off value = 0.05. (**B**) Centrality plots depicting the values of betweenness, closeness, strength, and expected influence centralities for every emotion. (**C**) Centrality measures per variable.

Before creating networks and studying them, the first stage of data analysis involved calculating the pairwise correlations between the six emotions for each individual per phase and for the whole process. Pearson analysis was carried out, and factor correlation matrices were formed for each participant, to study the possible correlations between the emotions in the experimental process. This analytical stage was performed using SPSS v.21, aiming to define the appropriate estimator in the creation of the emotion network in the next stage of the analysis. In the second stage, the JASP software (v.0.18.1.0) was used to create the emotion network. The mapping of the emotion network formed for each individual was carried out to reveal the dominant emotions. The correlation estimator was used for all the formed networks and “Cor” was used for the correlation method. The six emotions were the nodes of the formed network for each individual. The correlations between the nodes are shown in red and blue lines in Figure 5A, representing the negative and positive correlations, respectively. The role of each node in the network was determined by the centrality values, which were multiple and included the following:

- Betweenness [45]: Betweenness represents the number of times that a node is the intermediate connecting two other nodes, i.e., acting as a bridge node between them.
- Closeness [46]: Closeness defines the proximity of a node to other nodes in the network in terms of the number of direct and indirect connections (the average distance from the node to all other nodes in the network). The higher the magnitude of this centrality, the greater and more direct influence it has, or the more it is influenced by others in the network [47].
- Strength [48]: Strength identifies the weight of a node’s influence on others in the network. The higher the value of this centrality, the more central the role played by a node in the formed network.
- Expected influence [49,50]: The expected influence is the sum of all edges extending from a given node (maintaining the sign) while considering the presence of negative associations. It computes the node strength without taking the absolute value of the edge weights.

The participants were divided into three groups in terms of their achievement in the examination, depending on their final performance in the experimental process, reflected in the scores they received according to their success in the written and oral process. The first group consisted of twelve participants who demonstrated poor and moderate performance, the second consisted of twelve participants with good performance, and the third consisted of only two participants with excellent performance.

## 3. Results

The total number of volunteer participants in this study was originally thirty, but data from four of them were not used due to missing values resulting from disconnections in the EEG equipment. The emotion data for each subject were used to study the network formed via JASP software (v.0.18.1.0). Correlation was used as the estimator, and the correlation method was Cor. The formation of strong, mostly positive links (in most cases) was observed between them. The emotion of engagement, in most cases, did not significantly impact stress, excitement, focus, interest, or relaxation. The centralities proved that engagement played a minor role in the plot’s emotional network. Figure 5A,B shows an example of a person’s formed emotion network and centrality graphs. According to Figure 5C of centrality values, the emotion of stress showed closeness centrality, strength centrality, and expected influence centrality, with values of 0.857, 0.837, and 0.837, respectively. Excitement showed closeness centrality, strength centrality, and expected influence centrality, with values of 0.037, 0.115, and 0.115, respectively. Focus showed closeness centrality, strength centrality, and expected influence centrality, with values of −0.163, 0.028, and 0.028, respectively. Interest showed closeness centrality, strength centrality, and expected influence centrality, with values of 1.022, 0.773, and 0.773. Relaxation showed closeness centrality, strength centrality, and expected influence centrality, with values of 0.033, 0.162, and 0.162, respectively. Engagement showed closeness centrality, strength centrality, and expected influence centrality, with values of −1.786, −1.914, and −1.914, respectively. Engagement was the most distant node of the formed network and did not strongly correlate with the other emotions. No reference is made to betweenness centrality in the types of centralities presented in Figure 5C, as the network of agents consisted of only six nodes, which made it small. Moreover, no increased values for this centrality were observed, as the nodes were directly interconnected in this network, and no node was interposed between the connection of two others. The centrality graph shows that only the emotion of interest showed betweenness centrality (2.042) compared with the other emotions, which all had negative values (−0.408).

The overall results help us to understand the formed networks for all subjects involved in the experimental procedure. The data series for all subjects in this study are available upon request from the corresponding author for privacy and ethical reasons. The conclusions drawn from the emotion networks formed are important and relate to the following:

- Stress was, in most cases, the node with the most central role in the process, showing the highest values for closeness centrality (42.5%), strength (57.6%), and expected influence centrality (80.7%) compared with the other emotions.
- Stress had the strongest positive correlations with the other emotions compared with the other nodes in the network.
- If stress was removed as a node, the network, in most cases, appeared to be disordered, and the links between nodes were disrupted.
- The emotion of engagement had the lowest correlations with the other nodes and showed the lowest centrality values.
- The emotion of excitement showed centrality in the network after stress (closeness centrality = 15.3%, strength centrality = 11.5%, and expected influence centrality = 15.3%).
- The emotion of interest was most often the node interposed between two other connecting nodes, often showing a high betweenness centrality (34.6%).

## 4. Discussion

Studying emotions during a stressful process can produce interesting results. Important results were extracted from the analysis of the emotion network formed, comprising stress, interest, excitement, focus, relaxation, and engagement, with stress being the primary emotion. The dominant role of stress, induced via the research protocol, was confirmed via the centrality study. Its strong positive correlations with the other emotions were typical in most cases. According to the extracted data, stress showed even more centrality in the network during the oral examination, which was the most stressful phase of the experimental procedure. Therefore, the more elevated this emotion was, the more central the role that it played in the network.

Employing the network analysis method showed the interconnections between stress and the other measured emotions. Stress affected (and was affected by) interest, excitement, focus, relaxation, and engagement. Student performance was influenced by this status of complexity. To cope with stress and other emotions, the correlations between them are a key factor to consider. A certain amount of stress plays an imperative role in students’ examination performance. Furthermore, the levels of interest, excitement, focus, relaxation, and engagement could be turning points in transforming distress into eustress. Following this approach, educators may consider implementing methods during the learning process that inspire interest and enthusiasm for the courses they teach, raising attention and focus, to control stress and keep it within creative levels. Considering that the memory imprint is significantly related to the strength of the stimuli provided by the educator, learning methods could be facilitated by increasing interest, enthusiasm, and focus.

Previous research based on Bloom’s taxonomy demonstrated that students’ sense of interest can be enhanced via innovative synchronous and asynchronous teaching methods, such as flipped classrooms and the metaverse (the blended learning method (BLM)), with six student personas identified [51]. A different learning tool can be used for each persona, indicating a customized learning process according to the student’s personality. Other research with the same purpose reported that the flipped classroom method could enhance student engagement (especially when used alongside augmented reality [52,53]) and the emotion of focus [54]. Different techniques can be applied to enhance the emotion of relaxation from the instructors’ point of view, such as educational videos, brain games, communication enhancement, and wellbeing interventions [55]. Using active learning techniques (ALTs) enhances the emotion of excitement, improving student performance [56]. Using innovative teaching methods customized to each student persona can enable the emotions associated with stress to enhance student performance by transforming stress from a destructive into a creative emotion during the examination process, more effectively delivering the intangible knowledge acquired from the learning process. Stress is a dominant emotion during the stressful examination process, but its negative bodily effects can be alleviated by cultivating other emotions.

In a subsequent study, the data from this research may be considered from the perspective of econometric models and time-series analysis software, to observe the autocorrelation of the dominant emotion of stress and its cross-correlation with other emotions to study it like time-series data. This would also allow the brand of each course (the way students experience it, i.e., student perception) to be studied in relation to the emotions investigated in this paper and the students’ final performance.

## Data Availability

The data presented in this study are available on request from the corresponding author for privacy and ethical reasons.

## References

[1] Botinis A. (1989). Stress and Prosodic Structure in Greek.

[2] Fink G. (2016). Stress, Definitions, Mechanisms, and Effects Outlined. Stress: Concepts, Cognition, Emotion, and Behavior.

[3] Masi G., Amprimo G., Ferraris C., Priano L. (2023). Stress and Workload Assessment in Aviation—A Narrative Review. Sensors.

[4] Gardani M., Bradford DR R., Russell K., Allan S., Beattie L., Ellis J., Akram U. (2022). A systematic review and meta-analysis of poor sleep, insomnia symptoms and stress in undergraduate students. Sleep Med. Rev..

[5] Deng Y., Cherian J., Khan N.U.N., Kumari K., Sial M.S., Comite U., Gavurova B., Popp J. (2022). Family and Academic Stress and Their Impact on Students’ Depression Level and Academic Performance. Front. Psychiatry.

[6] Eisenberg D., Golberstein E., Hunt J. (2009). Mental Health and Academic Success in College. B.E. J. Econ. Anal. Policy.

[7] Bruffaerts R., Mortier P., Auerbach R.P., Alonso J., De la Torre A.E.H., Cuijpers P., Demyttenaere K., Ebert D.D., Green J.G., Hasking P. (2019). Lifetime and 12-month treatment for mental disorders and suicidal thoughts and behaviors among first year college students. Int. J. Methods Psychiatr. Res..

[8] Mortier P., Cuijpers P., Kiekens G., Auerbach R.P., Demyttenaere K., Green J., Kessler R.C., Nock M.K., Bruffærts R. (2017). The prevalence of suicidal thoughts and behaviours among college students: A meta-analysis. Psychol. Med..

[9] Karyotaki E., Cuijpers P., Albor Y., Alonso J., Auerbach R.P., Bantjes J., Bruffærts R., Ebert D.D., Hasking P., Kiekens G. (2020). Sources of Stress and Their Associations With Mental Disorders Among College Students: Results of the World Health Organization World Mental Health Surveys International College Student Initiative. Front. Psychol..

[10] Wunsch K., Fiedler J., Bachert P., Wöll A. (2021). The Tridirectional Relationship among Physical Activity, Stress, and Academic Performance in University Students: A Systematic Review and Meta-Analysis. Int. J. Environ. Res. Public Health.

[11] Campeau S., Liberzon I., Morilak D.A., Ressler K.J. (2011). Stress modulation of cognitive and affective processes. Stress.

[12] Kogler L., Müller V.I., Chang A., Eickhoff S.B., Fox P.T., Gur R.C., Derntl B. (2015). Psychosocial versus physiological stress—Meta-analyses on deactivations and activations of the neural correlates of stress reactions. NeuroImage.

[13] Berretz G., Packheiser J., Kumsta R., Wolf O.T., Ocklenburg S. (2021). The brain under stress—A systematic review and activation likelihood estimation meta-analysis of changes in BOLD signal associated with acute stress exposure. Neurosci. Biobehav. Rev..

[14] Selye H. (1956). The Stress of Life.

[15] Selye H., Cooper C.L. (1983). The stress concept: Past, present, and future. Stress Research.

[16] Giovanniello J., Bravo-Rivera C., Rosenkranz A., Matthew Lattal K. (2023). Stress, associative learning, and decision-making. Neurobiol. Learn. Mem..

[17] Cabeza R., Nyberg L. (2000). Imaging Cognition II: An Empirical Review of 275 PET and fMRI Studies. J. Cogn. Neurosci..

[18] Sousa D.A. (2022). How the Brain Learns.

[19] Lindau M., Almkvist O., Mohammed A.K. (2016). Effects of Stress on Learning and Memory. Stress: Concepts, Cognition, Emotion, and Behavior.

[20] Li X., Zhang Y., Tiwari P., Song D., Hu B., Yang M., Zhao Z., Kumar N., Marttinen P. (2022). Eeg based emotion recognition: A tutorial and review. ACM Comput. Surv..

[21] Suhaimi N.S., Mountstephens J., Teo J. (2020). Eeg-based emotion recognition: A state-of-the-art review of current trends and opportunities. Comput. Intell. Neurosci..

[22] Sharma R., Chopra K. (2020). Eeg signal analysis and detection of stress using classification techniques. J. Inf. Optim. Sci..

[23] Vanhollebeke G., Smet S.D., Raedt R.D., Baeken C., van Mierlo P., Vanderhasselt M.-A. (2022). The neural correlates of psychosocial stress: A systematic review and meta-analysis of spectral analysis EEG studies. Neurobiol. Stress.

[24] Niemic C. (2004). Studies of Emotion: A Theoretical and Empirical Review of Psychophysiological Studies of Emotion. J. Undergrad. Res..

[25] Katmah R., Al-Shargie F., Tariq U., Babiloni F., Mughairbi F.A., Al-Nashash H. (2021). A Review on Mental Stress Assessment Methods Using EEG Signals. Sensors.

[26] Hernández-Mustieles M.A., Lima-Carmona Y.E., Pacheco-Ramírez M.A., Mendoza-Armenta A.A., Romero-Gómez J.E., Cruz-Gómez C.F., Rodríguez-Alvarado D.C., Arceo A., Cruz-Garza J.G., Ramírez-Moreno M.A. (2024). Wearable Biosensor Technology in Education: A Systematic Review. Sensors.

[27] Allen A.P., Kennedy P., Dockray S., Cryan J.F., Dinan T.G., Clarke G. (2017). The Trier Social Stress Test: Principles and practice. Neurobiol. Stress.

[28] Chen H., Song Y., Li X. (2019). A deep learning framework for identifying children with ADHD using an EEG-based brain network. Neurocomputing.

[29] Li P., Liu H., Si Y., Li C., Li F., Zhu X., Huang X., Zeng Y., Yao D., Zhang Y. (2019). EEG based emotion recognition by combining functional connectivity network and local activations. IEEE Trans. Biomed. Eng..

[30] Sánchez-Reolid R., Martinez-Saez M.C., García-Martínez B., Fernández-Aguilar L., Ros L., Postigo J.M.L., Fernández-Caballero A. (2022). Emotion Classification from EEG with a Low-Cost BCI Versus a High-End Equipment. Int. J. Neural Syst..

[31] Schmittmann V.D., Cramer AO J., Waldorp L.J., Epskamp S., Kievit R.A., Borsboom D. (2013). Deconstructing the construct: A network perspective on psychological phenomena. New Ideas Psychol..

[32] Cramer A.O., Waldorp L.J., Van Der Maas H.L., Borsboom D. (2010). Comorbidity: A network perspective. Behav. Brain Sci..

[33] Epskamp S., Rhemtulla M., Borsboom D. (2017). Generalized network psychometrics: Combining network and latent variable models. Psychometrika.

[34] Žabčíková M. (2019). Measurement of visual and auditory stimuli using EEG headset emotiv Epoc+. MATEC Web Conf..

[35] Ergan S., Radwan A., Zou Z., Tseng H.A., Han X. (2019). Quantifying human experience in architectural spaces with integrated virtual reality and body sensor networks. J. Comput. Civ. Eng..

[36] Osornio García F.U., Fragoso González G.A., Martínez Pérez M.V., Báez Martínez F., Salas Barraza M.H., González V.M. (2023). Emotional analysis through EEG on in-store journey. HCI in Business, Government and Organizations.

[37] Williams N., McArthur G., Badcock N.A. 10 years of Epoc: A scoping review of emotiv’s portable EEG device. bioRxiv.

[38] Faruk MJ H., Valero M., Shahriar H. An investigation on non-invasive brain-computer interfaces: Emotiv Epoc+ neuroheadset and its effectiveness. Proceedings of the 2021 IEEE 45th Annual Computers, Software, and Applications Conference (COMPSAC).

[39] Ramírez R., Vamvakousis Z. (2012). Detecting emotion from eeg signals using the emotive Epoc device. Brain Informatics.

[40] Yurdem B., Akpinar B., Özkurt A. EEG data acquisition and analysis for human emotions. Proceedings of the 2019 11th International Conference on Electrical and Electronics Engineering (ELECO).

[41] Guo J., Smitha K.G. EEG based stress level identification. Proceedings of the 2016 IEEE International Conference on Systems, Man, and Cybernetics (SMC).

[42] Pham T.D., Tran D. (2012). Emotion recognition using the emotiv epoc device. Neural Information Processing.

[43] https://www.emotiv.com/pages/performance-metrics.

[44] https://www.emotiv.com/products/epoc.

[45] Barthélemy M. (2004). Betweenness centrality in large complex networks. Eur. Phys. J. B—Condens. Matter.

[46] Evans T.S., Chen B. (2022). Linking the network centrality measures closeness and degree. Commun. Phys..

[47] Borsboom D., Cramer A.O.J. (2013). Network Analysis: An Integrative Approach to the Structure of Psychopathology. Annu. Rev. Clin. Psychol..

[48] Bröhl T., Lehnertz K. (2022). A straightforward edge centrality concept derived from generalizing degree and strength. Sci. Rep..

[49] Robinaugh D.J., Millner A.J., McNally R.J. (2016). Identifying highly influential nodes in the complicated grief network. J. Abnorm. Psychol..

[50] Fonseca-Pedrero E., Ortuño J., Debbané M., Chan R.C.K., Cicero D.C., Zhang L.C., Brenner C.A., Barkus E., Linscott R.J., Kwapil T.R. (2018). The Network Structure of Schizotypal Personality Traits. Schizophr. Bull..

[51] Kilipiris F., Avdimiotis S., Christou E., Tragouda A., Konstantinidis I. (2024). Bloom’s Taxonomy Student Persona Responses to Blended Learning Methods Employing the Metaverse and Flipped Classroom Tools. Educ. Sci..

[52] Wen Y. (2020). Augmented reality enhanced cognitive engagement: Designing classroom-based collaborative learning activities for young language learners. Educ. Technol. Res. Dev..

[53] Huang Y.-M., Silitonga L.M., Wu T.-T. (2022). Applying a business simulation game in a flipped classroom to enhance engagement, learning achievement, and higher-order thinking skills. Comput. Educ..

[54] Hsieh J.C., Wu W.-C.V., Marek M.W. (2016). Using the flipped classroom to enhance EFL learning. Comput. Assist. Lang. Learn..

[55] Flinchbaugh C., Moore EW G., Chang Y.K., May D.R. (2011). Student Well-Being Interventions. J. Manag. Educ..

[56] Hendrickson P. (2019). Effect of Active Learning Techniques on Student Excitement, Interest, and Self-Efficacy. J. Political Sci. Educ..

